# Pan-Cancer Analysis Reveals a Distinct Neutrophil Extracellular Trap-Associated Regulatory Pattern

**DOI:** 10.3389/fimmu.2022.798022

**Published:** 2022-03-31

**Authors:** Xiao-Tian Shen, Sun-Zhe Xie, Jing Xu, Lu-Yu Yang, Lun-Xiu Qin

**Affiliations:** ^1^ Department of General Surgery, Huashan Hospital, Fudan University, Shanghai, China; ^2^ Cancer Metastasis Institute, Fudan University, Shanghai, China

**Keywords:** cancer, neutrophil extracellular traps (NETs), neutrophils, prognosis, tumor microenvironment (TME), SPP1

## Abstract

**Background:**

Neutrophils form extracellular net-like structures called neutrophil extracellular traps (NETs). Emerging evidence has shown that cancer can induce NET formation; however, it is not fully understood how NETs influence cancer biology, and no consensus has been reached on their pro- or antitumor effects. A comprehensive analysis of the global NET-associated gene regulatory network is currently unavailable and is urgently needed.

**Methods:**

We systematically explored and discussed NET enrichment, NET-associated gene regulatory patterns, and the prognostic implications of NETs in approximately 8,000 patients across 22 major human cancer types. We identified NET-associated regulatory gene sets that we then screened for NET-associated regulatory patterns that might affect patient survival. We functionally annotated the NET-associated regulatory patterns to compare the biological differences between NET-related survival subgroups.

**Results:**

A gene set variation analysis (GSVA) based on 23 major component genes was used to calculate a metric called the NET score. We found that the NET score was closely associated with many important cancer hallmarks, particularly inflammatory responses and epithelial-to-mesenchymal transition (EMT)-induced metastasis. Higher NET scores were related to poor immunotherapy response. Survival analysis revealed that NETs had diverse prognostic impacts among various cancer types. The NET-associated regulatory patterns linked to shorter or longer cancer patient survival were distinct from each other. Functional analysis revealed that more of the NET-associated regulatory genes linked to poor cancer survival were associated with extracellular matrix (ECM) remodeling and pan-cancerous risk factors. SPP1 was found to be highly expressed and correlated with NET formation in cancers with poor survival. We also found that the co-upregulation of NET formation and SPP1 expression was closely linked to increased EMT and poor survival, that SPP1 influenced NET-induced malignant capacity, and that SPP1 overproduction induced a robust formation of metastatic-promoting NETs.

**Conclusion:**

NETs were common across cancers but displayed a diverse regulatory pattern and outcome readouts in different cancer types. SPP1 is potentially the key to NET-related poor outcomes.

## Introduction

Neutrophils are the most abundant circulating innate immune cells, mainly participating in killing bacteria, fungi, parasites, and viruses in infectious diseases (1). Neutrophils also play important roles in other conditions, such as autoimmune, skeletomuscular, and vascular diseases, as well as in cancer (2–5). Neutrophils exert their effects by undergoing NETosis, which starts with cellular and nuclear breakdown, followed by the release of cytosolic and granule proteins and DNA, ultimately leading to the formation of large, extracellular net-like structures called neutrophil extracellular traps (NETs) (6–8). The range of functions of NETs is complicated, and major NET-related pathologies include cell damage, inflammation, vaso-occlusion, and autoimmunity.

NET formation is dysregulated in cancer. Evidence shows that tumor-secreted factors, including G-CSF, IL8, and exosomes (9, 10), lead to granulocyte accumulation and subsequent NET formation. It remains controversial whether NET formation is a friend or a foe. NETs were first identified as a contributor to cancer-associated thrombosis (11), and further studies have highlighted the critical roles of NETs in cancer progression, coagulation, and metastasis (12). Our previous work and works of others suggest that NETs provide proximal scaffold structures for passive adhesion or chemotaxis of metastatic tumor cells in liver, colon, and breast cancer (8, 13, 14). NETs subsequently stimulate metastasis by provoking tumor-associated inflammatory responses, by awakening quiescent cells, or by maintaining mitochondria homeostasis (8, 15, 16). Moreover, NETs form a coat around tumor cells to protect them from immune cytotoxicity (17, 18). However, opposite trends have also been reported. In high-grade ovarian cancer, higher levels of NET-related proteins in ascites is correlated with favorable prognosis (19); furthermore, infiltrated NETs can inhibit cell migration in melanoma, and *in vivo* coculture of NETs and melanoma can stimulate necrosis (20). In addition, NETs can cause *in situ* cytotoxic-related cell damage in bladder cancer, thereby facilitating improved bacillus Calmette–Guerin treatment outcomes (21). The functional roles of NETs (and the associated underlying mechanisms) in tumors and tumorous environments, especially in terms of their various roles across different cancer types, require further exploration.

In this study, we evaluated the NET level in various cancer types in The Cancer Genome Atlas (TCGA) using gene set enrichment analysis (GSEA) scores of NET-associated gene signatures curated from previous studies (3, 22). A functional analysis revealed a close relationship between cancer hallmarks and NET score. Furthermore, two distinct NET-associated regulatory patterns were identified in cancers, one with a favorable prognostic impact and one with a poor prognostic impact. The current study provides a comprehensive understanding of the regulatory capacity of infiltrated NETs, thus enabling further investigation of novel therapeutic approaches and methods for precise patient stratification.

## Materials and Methods

### Data Collection

FPKM-normalized mRNA expression profiles and clinical features of approximately 10,000 patients across 22 human cancer types were downloaded from TCGA data portal (https://portal.gdc.cancer.gov/, **Table S1**). An integrated PanCan TCGA expression set comprising approximately 10,000 samples was downloaded from Xena (UCSC Xena (xenabrowser.net)) after batch-effect removal. The BRCA protein expression profiles were downloaded from CPTAC (https://cptac-data-portal.georgetown.edu/cptac/s/S015). The gene expression arrays and responses to PD1 therapy for 2 bladder cancer samples and 1 skin cancer sample were downloaded from the GEO dataset with accession numbers GSE78220 and GSE91061 or from GitHub (https://github.com/hammerlab/multi-omic-urothelial-anti-pdl1).

### Analysis of NET Profiles in Cancers

FPKM-normalized RNA-seq expression counts were downloaded from UCSC Xena (http://xena.ucsc.edu/). Only solid malignancies with sample sizes >50 tumor tissues and >10 normal tissues were enrolled, and, in total, 22 types of solid tumors were included for further analyses. A reference NET signature comprising 23 genes was derived using proteins enriched in NETs released from human neutrophils identified in previous studies (3, 4, 22).

The correlation between protein and RNA expression levels was validated using a Pearson correlation between RNA-seq and spectrometry data in matched BRCA samples in TCGA and CPTAC. The NET score, which was used to quantify the enrichment of NETs in tumors, was calculated *via* gene set variation analysis (GSVA) with the GSVA package (23). This analysis is based on a non-parametric and unsupervised method that is commonly used to estimate variation in the activities of pathways and biological processes in samples from expression datasets. The NET scores were grouped by quantiles into 4 groups for categorization and further analysis. Differential gene expression (DEG) analysis *via* the Limma package was used to explore expression differences in the NET profiles between tumor and para-tumor tissues. DEG analysis was performed using a PanCan dataset and for each individual cancer dataset.

### Evaluation of the Association Between NETs and Cancer Hallmarks

To minimize batch effect, we performed this analysis in separate cancer types instead of performing a pan-cancer analysis. Cancer hallmark gene sets, i.e., angiogenesis, anti-apoptosis, aneuploidy, EMT, glycolysis, hypoxia, inflammatory response, and proliferation, were downloaded from MSigDB (http://www.gsea-msigdb.org/gsea/msigdb). The association between cancer hallmarks was estimated by the Pearson correlation between the ssGSEA enrichment score of each gene set and the NET score. We considered |R| values >0.3 and p values <0.05 to be significant.

### Evaluation of the Association Between NETs and Tumor Microenvironment Alterations

MCPcount (24) was used to derive the enrichment estimate of infiltrated immune and stromal cells in each individual cancer data set. The association between infiltrated cells and NETs was estimated by the Pearson correlation between the MCPcount value of each cell and the NET score. We considered |R| values >0.3 and p values <0.05 to be significant.

Chemotaxis-related gene sets from B cells, dendritic cells, endothelial cells, eosinophils, granulocytes, leukocytes, lymphocytes, macrophages, monocytes, and natural killer cells were downloaded from MSigDB. For each individual cancer types, the gene sets were compared between the highest 25% and the lowest 25% groups of NETs, using (fold change) FC >2 and p value <0.05 as the threshold for significance. For visualization, the immune cells, rather than genes, were labeled to make results more straightforward.

### Survival Analysis of Cancer Patients With Different NET Scores

Patients with each cancer type were stratified into high- or low-NET score groups using a cutoff value determined using the survival package. Kaplan–Meier curves, log-rank tests, and single variate Cox estimate models were used to evaluate the prognostic difference between the high- and low-NET score groups. The cancer types with favorable survival in the high-NET group (log-rank p test <0.05, HR >1) were considered cancer types with favorable NET-related survival, while cancer types with poor survival in the low-NET group were considered cancer types with poor NET-related survival. An overall effect of NETs on pan-cancer survival was estimated in the PANCAN dataset *via* Kaplan–Meier analysis of the quantile-stratified NETs scores.

A similar analysis was performed to investigate the survival impact of NET-associated regulatory genes. We defined a set of NET-associated regulatory genes using a co-expression pattern. Typically, in an individual cancer type, genes with Pearson correlation R values >0.35 or <-0.35 and p values < 0.05 were marked as NET-associated regulatory genes. To shorten the gene list and to identify a universal NET-associated regulatory pattern, we filtered out the NET-associated regulatory genes that appeared in less than 5 cancer types in cancer subgroups with poor or favorable NET-related survival. This processing left approximately 500 NET-gene pairs in cancer types with favorable or poor NET-related survival.

### Evaluation of the Effect of NETs on Immune Therapy Response

Three PD1 inhibitor treatment cohorts (25–27) were included to estimate the influence of NETs on immune therapy outcomes. The patients were categorized into response (partial response/complete response/stable disease) or non-response groups according to RECIST criteria. NET scores were calculated and compared using the mRNA expression data for the two groups to evaluate the association between NETs and therapy response.

### Functional Gene Set Annotation

KEGG and GOBP gene sets were downloaded from the MSigDB database to functionally annotate the gene sets. Adjusted p values <0.05 were considered statistically significant. The “clusterProfiler” R package was used to perform functional annotation with a cutoff value of FDR <0.05. For gene set enrichment analysis (GSEA), the R package “GSVA” was used. The “enrichplot2” package was used for plotting. Adjusted p values <0.05 were considered significant.

### Cell Culture and Gene Knockdown

Human-derived HepG2 and MHCC97H hepatocellular carcinoma (HCC) cells, as well as mouse-derived Hepa1-6 HCC cells, were cultured in DMEM with 10% fetal bovine serum and 1% penicillin/streptomycin. For SPP1 knockdown, an SPP1-directed siRNA with the sequence 5-GCAUUCCGAUGUGAUUGAUtt-3;3-AUCAAUCACAUCGGAAUGCtt-5 was synthesized (www.biosune.com). The siRNA was prepared at a final concentration of 10 nM and transfected into cells using Lipo2000 (Hanhen bio https://www.hanbio.net/). Gene knockdown efficiency was monitored *via* qPCR and Western blotting.

For qPCR, total RNA was extracted using TRIzol (Invitrogen, Carlsbad, CA, USA) and reverse-transcribed into single-stranded cDNA using the PrimeScript™ RT Reagent Kit (TaKaRa Biotechnology, Shiga, Japan). Quantitative real-time PCR was performed with SYBR Green qPCR Master Mix (DBI Bioscience, Newark, DE, USA). The following SPP1-specific primers were used: F: CTCCATTGACTCGAACGACTC; R: CAGGTCTGCGAAACTTCTTAGAT. Expression levels were normalized against GAPDH in each sample and then normalized as fold change.

For Western blotting, protein was extracted using RIPA lysis buffer (P0013B, Beyotime, Shanghai, China, https://www.beyotime.com/). The cell lysates were separated in 10% gels (PG112, Epizyme, Suzhou, China, http://www.epizyme.cn/), transferred to polyvinylidene fluoride membranes, and probed with antibodies according to the manufacturer’s instructions. The following antibodies were used: SPP1 (Proteintech, Wuhan, China, 22952-1-AP) and GAPDH (Proteintech, Wuhan, China, 22952-1-AP).

### Construction of SPP1 Overexpression Cell Lines

HEK 293T embryonic kidney cells and mouse Hepa1-6 HCC cells were purchased from the Institute of Biochemistry and Cell Biology, Chinese Academy of Science (Shanghai, China).

Mouse SPP1 isoform 4 (NM_001204233.1) cDNA was amplified *via* PCR and cloned downstream of the tag in the pCDH-ZsGreen lentivirus vector. Primer sequences were as follows: F: 5′-cta gag gat cta ttt ccg gtg aat tca tga gat tgg cag tga ttt gct-3′ and R: 5′-tca ctt aag ctt ggt acc gag gat cct tag ttg acc tca gaa gat gaa ctc t-3′. The plasmid was transfected into cells using lentivirus. Stable cell lines were obtained *via* puromycin selection for at least 1 week.

### Orthotopic Implantation Model

Six-week-old male C57BL/6 mice were purchased from GemPharmatech, Jiangsu, China, and housed in a pathogen-free vivarium under standard conditions. All animal experiments were performed following the guidelines for the care and use of laboratory animals and were approved by the institutional review board of the Department of Laboratory Animal Science, Fudan University (Shanghai, China).

In each animal experiment, mice were randomly assigned to each group. Subcutaneous xenograft models were established *via* subcutaneous injection of 4 × 10^6^ Hepa1-6^spp1^ cells or Hepa1-6^CON^ cells into the right flanks of mice. Mice were sacrificed 20 days later, and the tumors were paraffin-embedded and sectioned.

### Immunofluorescent Tissue Staining

Immunofluorescent staining of NETs in paraffin-embedded sections was performed *via* the avidin–biotin–peroxidase complex method. Briefly, after rehydration, antigen retrieval, and elimination of autofluorescence, the samples were incubated in primary antibodies, H3cit (1:150, Abcam, Cambridge, MA, USA) and MPO (1:100, Abcam), overnight at 4°C. The samples were then incubated with fluorochrome-conjugated secondary antibodies and Hoechst 33342 for nucleus staining.

### Neutrophil Chemotaxis

Neutrophils were isolated from the blood obtained from HCC patients and healthy donors by a widely used one-step gradient centrifugation method using Polymorphprep (Axis-Shield, Dundee, UK) according to instruction and maintained in RPMI 1640 supplemented with 5% fetal calf serum (FCS) for immediate use. A purity of 90% was confirmed by flow cytometry using anti-CD15 antibody staining (BD Biosciences, Franklin Lakes, NJ, USA), with a viability rate over 95% by Trypan blue exclusion. Neutrophil chemotaxis was assayed in a Transwell system using 5-μm polycarbonate membranes as previously described (28). Briefly, rhOPN (1 µg/ml) was added to the lower chamber of the Transwell system in 2% FBS. Live cell dye Dil-labeled neutrophils (1 × 10^5^) were added to the upper chamber followed by incubation for 2 h. Neutrophils that migrated to the lower chamber were collected and counted in Neubauer chambers.

### NET Preparation

Human neutrophils were seeded in 6-well plates (1 × 10^7^/well) and stimulated with 20 nM PMA (phorbol 12-myristate 13-acetate) for 4 h. Next, without disturbing the NETs, the supernatants were carefully removed using slow suction, and the wells were washed twice to eliminate residual PMA or NET-unassociated substances. To digest the NETs, RPMI (1 ml) containing MNase (1 U/ml) was then added followed by incubation at 37˚C for 20 min. The nuclease activity was then stopped *via* treatment with 5 mM EDTA. The NET-containing supernatant was collected and centrifuged to eliminate cellular debris. The isolated NETs were stored at -80°C for future use.

### *In Vitro* Invasion Assays

1 × 10^5^ NET-stimulated cancer cells in serum-free DMEM were seeded in the upper chamber of an 8-μm Transwell system. The upper chamber of the Transwell system was precoated with 20% matrix gel. After incubation for 30 h, the contents of the upper chambers were collected. The cells on the lower membranes were then stained with crystal violet. Cells that migrated through the membrane to the lower surface were quantified in 4 random fields of view, and the data are reported as the fold change.

### Statistical Analysis

Correlation coefficients were computed as Pearson correlations with R. Survival analysis and Kaplan–Meier curves were generated with the “survival” R package, and log-rank tests were performed to examine significance. We adopted a univariate COX regression model to compute the hazard ratios (HR) with the “survival” R package. All statistical p values were two-tailed with p < 0.05 set as statistically significant. All data processing was done in R 3.6.3 software.

## Results

### Definition of a Pan-Cancer NET-Associated Dysregulation Profile

To generate an overview of NETs across different cancer types, we defined a pan-cancer NET signature of 23 genes in 22 cancers (**Table S1**). NET genes were identified as key NET components or as NET-driving factors in previous reports. Only solid tumors were enrolled (see *Methods*).

To quantify infiltrated NETs, we calculated the GSVA enrichment score of the NET signature (NET score) for each cancer type. Pearson correlation scores between the mRNA and protein levels of the NET genes in a CTPAC dataset revealed a moderate correlation for most NET genes (**Figure S1**). Notably, the key gene related to NET formation, MPO, showed the highest R value (0.51).

**Figure 1:**
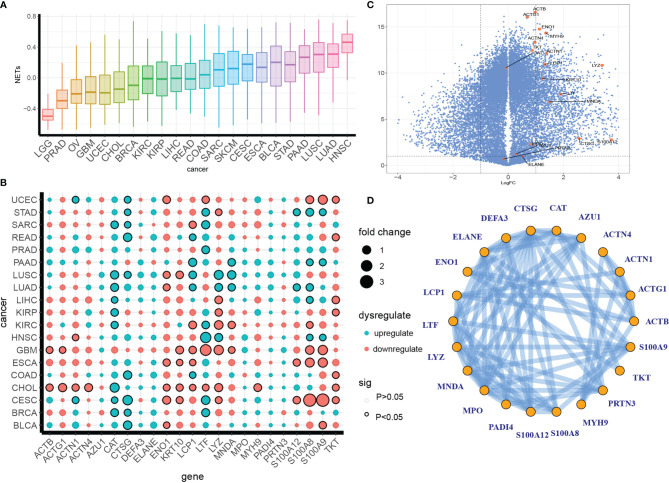
Definition of a pan-cancer NET dysregulation profile. **(A)** A pan-cancer GSVA enrichment score profile of NETs. **(B, C)** Irregular expression patterns of NETosis genes in separate cancer types **(B)** or pan-cancer **(C)**. The colors indicate up- or downregulation, and the dot size indicates the fold difference between tumor tissue and para-tumor tissue. The circle outlines indicate significance. **(D)** Protein–protein interactions of NETs proteins from the STING database.

The NET scores varied broadly across the different tumor types (**Figure 1A**), with head and neck cancer (HNSC) and two types of lung cancer (LUAD, LUSC) emerging as the top three cancer types for NET enrichment. Notably, despite coming from the same organ (but representing different malignancy types), low-grade glioma (LGG), which showed the least enrichment, had a much lower enrichment than glioblastoma (GBM). This result is consistent with a recent observation that a high level of neutrophil infiltration and NET formation are associated with high-grade glioma compared with low-grade glioma (29).

We compared the expression levels of NETs-associated genes between tumorous and para-tumor tissues in the PANCAN data set and individual cancer types and found dysregulation patterns that varied across cancer types and genes. CAT, CTSG, LTF, LCP1, S100A8, and S100A9 were the most dysregulated. LUSC, LUAD, GBM, ESCA, CHOL, and CESC showed the highest accumulation of dysregulated NET genes (**Figure 1B**). Furthermore, a volcano plot showed that most NET-associated genes were upregulated in a pan-cancer analysis (**Figure 1C**). NET formation is a complexly regulated process involving a series of biological steps and genetic interactions; therefore, we decided to investigate the co-interaction pattern of NET genes. To this end, we examined the potential protein–protein interaction network (PPI) of NET genes in the STING database and found a complex interaction network, indicating a co-regulatory network formed by NETs (**Figure 1D**).

Taken together, these results revealed a dysregulation pattern of NET formation in various cancer types.

### NET Score Is Closely Associated With Major Cancer Hallmarks, Especially Inflammatory Responses

**Figure 2:**
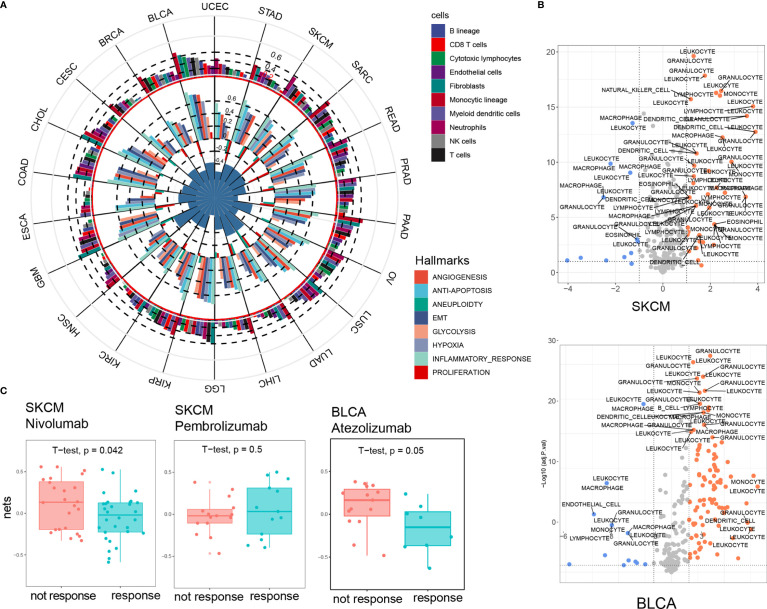
NET score is closely associated with major cancer hallmarks, especially inflammatory response. **(A)** NET score is strongly correlated with cancer hallmarks (inner circle) and immune infiltrating cells (outer circle). The circle diameter corresponds to the Pearson correlation value. **(B)** Chemotaxis genes are strongly differentially expressed in the high and low NET score groups. SKCM and BLCA are presented as examples. **(C)** Higher NET scores predict a poorer response to immune checkpoint blockade therapy.

We further explored the relationship between NET score and several key cancer hallmarks, including angiogenesis, antiapoptosis, aneuploidy, epithelial mesenchymal transition (EMT), glycolysis, hypoxia, inflammatory responses, and cell proliferation. We compared the Pearson correlations between the NETs and hallmark scores, which were represented by the ssGSEA scores of the gene sets. Six out of eight hallmarks showed a positive correlation with NET score. Among this group, inflammatory responses showed the highest correlation, with R values over 0.6 in 14 out of 22 cancer types. This finding is consistent with the results of our previous unbiased RNA-seq analysis, which showed that NETs trigger a tumor-associated inflammatory response in hepatocellular carcinoma (8). By contrast, proliferation and aneuploidy were negatively correlated with NET score in 20 cancer types (**Figure 2A**). In addition, HALLAMRKs, a well-defined biological state or process gene signature set, was also included in this analysis. Among the included processes, NF-κB signaling, KRAS signaling, and the complement response were highly correlated with NET score in most cancer types (**Figure S2**). These results indicated that NETs are associated with several key cancer-related biological processes.

Using MCPcounters, we deconvolved 10 immune and stromal cell components and found a positive association between NET score and monocytes, neutrophils, and myeloid dendritic cells in most cancer types; however, we found no significant association between NET score and cytotoxic T cells, NK cells, or B cells (**Figure 2A** and **Figures S3A–C**). We also compared the chemotaxis factor profiles of several types of immune cells, including leukocytes, granulocytes, and monocytes, between the NET-high and NET-low groups for each cancer type. A volcano plot showed that the NET-high group had generally higher expression levels of chemotaxis factors and a potentially stronger chemotaxis effect for granulocytes and leukocytes in all cancer types, indicating the existence of a complex regulatory network between NETs and immune cells in cancer (**Figure 2B** and **Figure S4**). In addition, a correlation analysis between NET score and 45 immune checkpoint genes (ICGs) revealed that CD48, CD86, and LAIR1 were the most significant NET-related checkpoint genes in all cancer types (**Figure S3D**). We then compared the NET score between the responsive group (PR, CR) and the non-responsive group (SD, PD) in 3 independent cohorts treated with anti-PD1 therapy (25–27). Consistent with the correlation between NET score and immune checkpoint genes, the NET scores were significantly higher in the non-responsive group in 2 out of 3 cohorts (**Figure 2C**). Taken together, these findings highlighted NETs as a central regulator of key cancer-related biological process and tumor-related immunology and suggest that NETs might be predictive of immune checkpoint inhibitory outcomes.

### NETs Have Diverse Prognostic Impact Across Different Cancer Types

**Figure 3:**
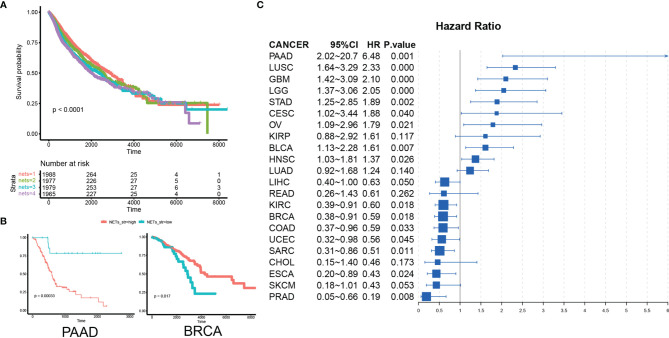
NETs have diverse prognostic implications in different cancer types. **(A)** A pan-cancer Kaplan–Meier curve showing survival associated with quantile-stratified NETs scores. **(B)** A Kaplan–Meier curve showing survival associated with stratified NETs scores in PAAD and BRCA. **(C)** A forest plot showing the hazard ratio of NETs score in cancer.

To investigate the prognostic impact of the NET score in cancer, we analyzed data from 8,059 patients from 22 different cancer types. These patients were stratified into 4 categories according to the NET score, and their survival was compared *via* Kaplan–Meier analysis. Cancer patients in the top quantile for NET score had the best prognosis, while patients in the bottom quantile had the poorest prognosis (**Figure 3A**). As the selected cancer types comprised very different types of malignancies, we then assessed the NET-related survival for each cancer type individually. In prostate cancer (PAAD), esophageal cancer (ESCA), skin cancer (SKCM), sarcoma (SARC), breast cancer (BRCA), and colon cancer (COAD), higher NET scores were related to favorable survival. By contrast, in pancreatic cancer (PDAC), lung squamosal cancer (LUSC), glioblastoma (GBM), low-grade glioma (LGG), ovarian cancer (OV), gastric cancer (STAD), and bladder cancer (BLCA), higher NET scores were associated with poorer survival (**Figures 3B, C**, **S5**). These results suggest the existence of two NET-related survival patterns in different cancer types.

### Identification of NET-Associated Regulatory Patterns in Cancers With Favorable or Poor Survival

**Figure 4:**
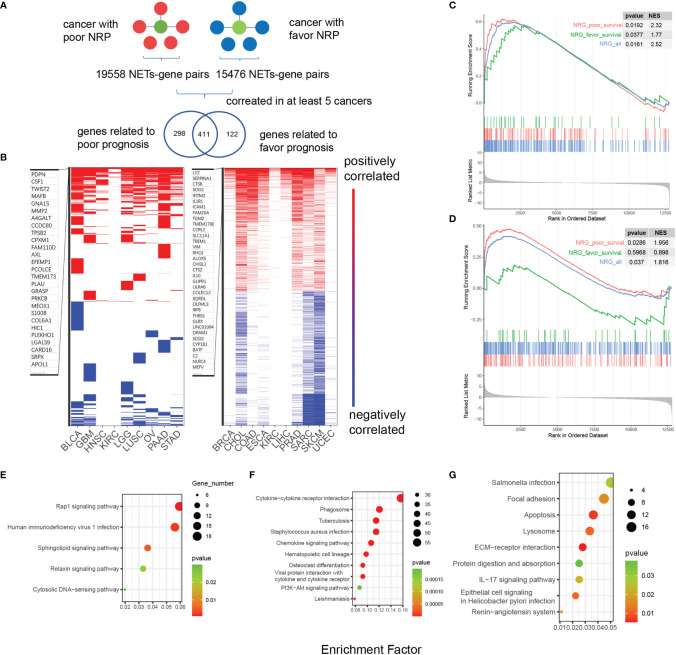
Identification of NET-associated regulatory patterns in cancers with poor or favorable NET-related survival. **(A)** A diagram showing the identification of NET-related genes. **(B)** A heatmap showing the correlated NET-related genes in cancer types with poor or favorable NETs-related survival. Red and blue bars indicated Pearson correlation values >0.4 or <-0.4, respectively. **(C, D)** GSEA showing the enrichment of two NET-related genes sets in HepG2 and MHCC97H cells after NET stimulation. **(E–G)** KEGG functional annotation of gene sets related to favorable **(E)**, risk **(G)**, or both **(F)** NET-related survival.

To explain the diverse NET-related outcomes in different cancer types, we defined groups of NET-related genes (NRG) that were strongly correlated with the NET score (Pearson R values <-0.35 or >0.35) for each cancer type. In total, 35,034 NET–NRG pairs were identified, among which 15,476 belonged to cancers with favorable NET-related survival and 19,558 belonged to cancers with poor NET-related survival (**Figure 4A**). The NET–NRG pairs identified in more than 5 cancer types in each survival group were further defined as core NET–NRG pairs, leaving 533 NET–NRG pairs in cancer types with favorable NET-related survival and 709 in cancer types with poor NET-related survival. In addition, 411 pairs overlapped (**Figure 4B**). We used a GSEA algorithm to compare the enrichment of 2 NRG sets in cancer types with favorable or poor NET-related survival. For the 122 NET–NRG pairs only found in cancer types with favorable NET-related survival, the normalized enrichment score (NES) was -1.1 (p = 0.04). For the 298 NET–NRG pairs only found in cancer types with poor NET-related survival, the NES was 2.27 (p < 0.001). These findings suggest the existence of different NRG enrichment patterns in cancer types with poor or favorable NET-related survival (**Figures S6A, B**). To further explore NRG dysregulation, we reexamined RNAseq data of HepG2 and MHCC97H cancer cells with and without NET stimulation from our previous study (8). This analysis revealed that NRGs with favorable or poor survival as well as all NRGs were upregulated after NET stimulation (**Figures 4C, D**). The NES scores for all NRGs in NET-stimulated HepG2 cells or MHCC97H cells were 2.32 (p = 0.0192) and 1.816 (p = 0.037), respectively. For the NRGs in cancer types with poor NET-related survival, the NES scores were 2.52 and 1.956 (p = 0.0161, 0.0286), while for cancer types with favorable NET-related survival, the NES scores were 1.77 and 0.838 (p = 0.0377, 0.5968).

Two sets of NET–NRG pairs had distinct biological functions. KEGG annotation of 2 sets of NET–NRG pairs showed that for NET–NRG pairs in cancer types with favorable NET-related survival, Rap1, sphingolipid signaling, and relaxin signaling were enriched. For NET–NRG pairs in cancers with poor NET-related survival, focal adhesion and ECM–receptor interactions were enriched. For shared NET–NRG pairs, cytokine-related pathways and *Staphylococcus aureus* infection were jointly enriched (**Figures 4E–G**). Based on these combined data, we identified and experimentally validated two different NET-associated regulatory gene patterns in cancer. These patterns were differentially enriched in cancer types with favorable or poor NET-related survival, and they differed in their underlying biological functions. We propose that these patterns might crucially influence NET-related survival.

### NET-Associated Regulatory Patterns Are Linked to Prognosis-Related, Co-Expressed Gene Clusters

**Figure 5:**
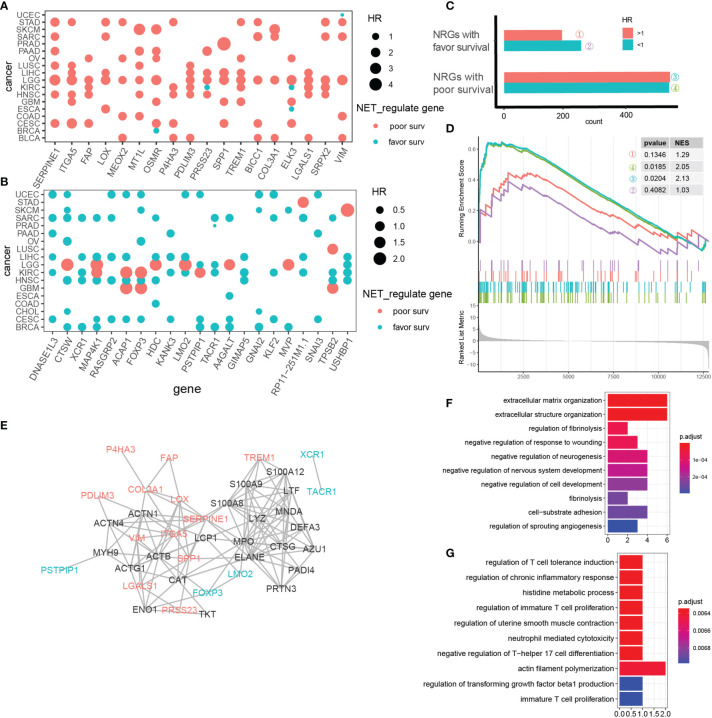
NET-associated regulatory patterns comprise prognosis-related, co-expressed gene clusters. **(A, B)** NET-associated regulatory genes with a pan-cancerous risk prognosis **(A)** or favorable prognosis **(B)**. Red indicates risk genes, while blue indicates favorable genes. Dot size represents the HR value. **(C)** Cox model HR estimates for mean stratified NET-associated regulatory genes with p value <0.05. **(D)** GSEA showing upregulation of 4 categories of NET-associated regulatory genes defined in **(C)** in MHCC97H cells under NET stimulation. **(E)** A protein–protein network showing the interactions between 18 NRGs related to pan-cancer risk prognosis (red); 22 NRGs related to pan-cancer favorable prognosis (blue), and 23 NETs genes (black). Only genes with at least one interaction are shown. **(F)** KEGG pathway analysis of NET-associated regulatory genes related to pan-cancer risk prognosis. **(G)** KEGG pathway analysis of NET-associated regulatory genes related to pan-cancer favorable prognosis.

We hypothesized that NET-related genes comprised 2 groups: those with pro-tumor effects and those with antitumor effects. To explore this possibility, we assessed the individual expression bias of NRGs in various cancer types and determined the overall prognostic impact of the NET scores. We calculated the hazard ratios (HRs) of NRGs using a univariate Cox model with the mean values of the NRGs as a cutoff. A total of 9,270 models were fit, of which 1,578 were significant (p < 0.05). We plotted the HR distribution of the NRGs and allocated the NRGs to groups of poor or favorable prognostic markers in a pan-cancerous context. NET-associated regulatory genes in cancer types with a poor NET-related survival tended to be risk-decisive, such as LGG and GBM (**Figure S6** and **Figures 5A, B**). Among the 298 NRGs associated with cancer types with poor survival, 559 and 562 models were fit with HR >1 or <1 (p < 0.05), while in 122 NRGs with favorable survival, additional models with HR <1 were fit (261 vs. 196 models with HR >1) (**Figure 5C**). We validated the NRG expression alterations in RNAseq data reflecting the transcriptome of HepG2 cells before and after NET stimulation, and we proved that these genes were upregulated after NET stimulation (**Figure 5D**). We applied STRING analysis to 18 NRGs related to poor pan-cancer prognosis, 22 NRGs related to favorable pan-cancer prognosis, and 23 NET score genes (**Figure 5E**). Only genes with at least one interaction were retained. A total of 135 gene–gene interactions were identified in the STRING database. It turned out that NETs have a closer association with NRGs related with poor prognosis instead of those related with good prognosis. Hub gene analysis using cytoHubber revealed MPO, CTSG, and LYZ as the hub genes with the most gene–gene interactions.

To further explore the biological functions of these genes, we performed KEGG annotation. The top 10 enriched pathways sorted by p value were shown (**Figures 5F, G**). For NET-associated regulatory genes related to poor pan-cancer prognosis, extracellular matrix-modulating pathways were the most relevant. For NET-associated regulatory genes related to favorable pan-cancer prognosis, surprisingly, T-cell immunity regulation dominated.

In this work, we categorized NET-associated regulatory genes according to their pan-cancerous prognostic impact and identified 2 clusters of genes that 1) were linked to global poor or favorable survival and 2) showed an expression bias between the two survival groups. These gene clusters represented divergent biological functions, indicating that NETs may play both pro- and antitumor roles by regulating different biological processes.

### Identification of SPP1 as a Key NET-Related Gene That Facilitates NET-Related Malignancy

**Figure 6:**
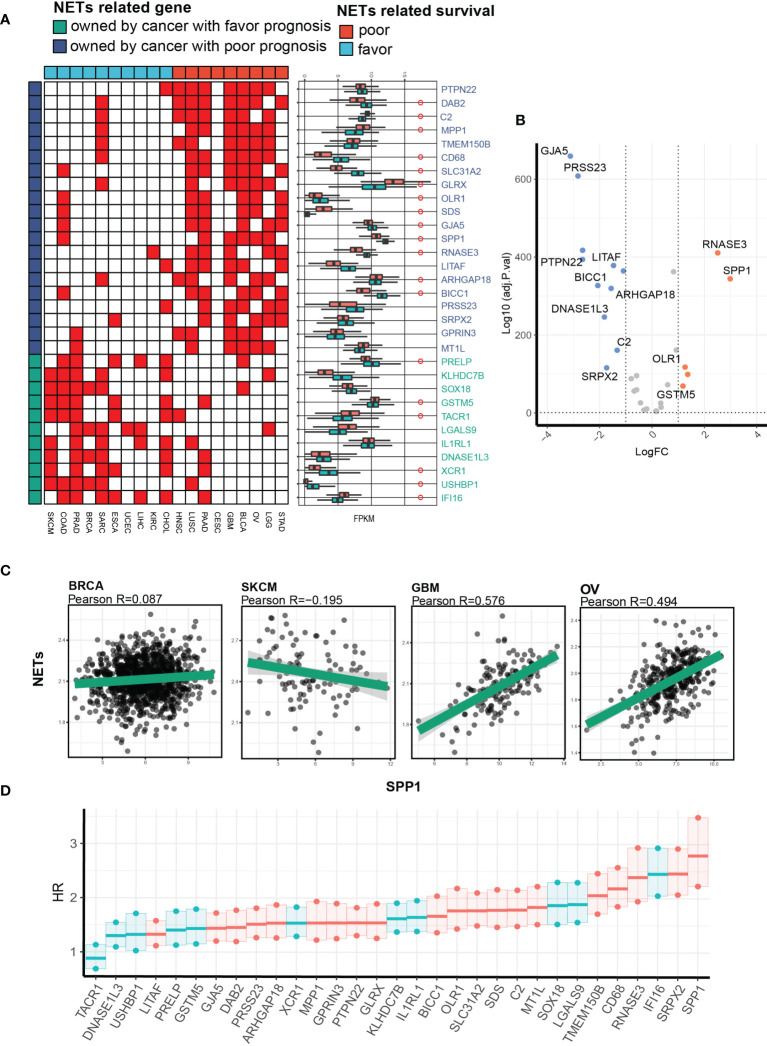
Identification of SPP1 as a key NET-related gene facilitating NET-related malignancy. **(A)** A heatmap showing the NET-related genes with the highest correlation and differential expression between cancers with favorable or risk NET-related survival. **(B)** A volcano plot showing expression differences. **(C)** Representative Pearson correlation values between NET score and pan-cancerous SPP1 expression. **(D)** A univariate Cox proportional hazard model showing the hazard ratios for death in the NETs_high_gene_high group compared with the NETs_low_gene_low group in a pan-cancer cohort.

We narrowed down the number of gene–NET pairs by retaining only pairs with positive correlation >5 in sets of cancer types with favorable/poor NET-related survival and positive correlation <2 in another set of cancer types. For example, PTPN22 was positively correlated with NETs in 7 cancer types with poor NET-related survival and in 1 cancer type with favorable NET-related survival. After this analysis, we identified 20 and 11 NRGs with Pearson R values >0.4 in cancer types with favorable or poor NET-related survival (**Figure 6A**). We compared the gene expression levels between the two cancer groups and defined a set of genes universally related to NETs, among which SPP1 was highly expressed in cancers with poor NET-related survival (fold change of 2.3) (**Figure 6B**). The Pearson R value between SPP1 and NET score showed that SPP1 had a positive correlation with NET score only in cancer types with poor NET-related survival (GBM, R = 0.576; OV: R = 0.494). In cancer types with favorable NET-related survival, no correlation was found (BRCA: R = 0.087; SKCM: R = -0.195) (**Figure 6C**). We further explored the integrated effect of genes and NET score on patient outcomes using a univariate Cox model. We identified a significant survival difference between different subgroups in which SPP1/NETs-high patients had a poorer prognosis (HR = 2.75) (**Figure 6D**). Collectively, we found that SPP1 was highly expressed and correlated with NET score in cancer types with poor NET-related survival.

### A Potential Cooperative Pro-Tumor Effect of SPP1 and NETs

**Figure 7:**
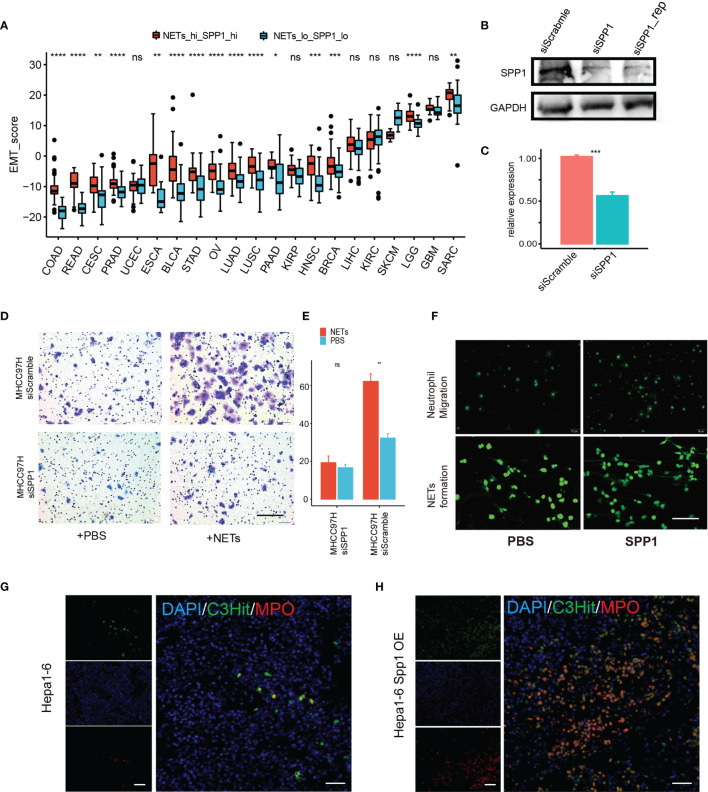
A potential cooperative pro-tumor effect of SPP1 and NETs. **(A)** EMT score differences between the NET score top—SPP1 top subset and the NET score bottom—SPP1 bottom subset for each tumor type. **(B, C)** Western blot **(B)** and qPCR **(C)** results showing successful siRNA knockdown of SPP1 in MHCC97H cells. **(D, E)** Transwell assay measuring the migration ability of MHCC97H cells with/without NET stimulation and with/without SPP1 knockdown. Scale bar:50 µm. **(F)** Exogenesis treatment with SPP1 protein (10 ng/ml) promotes neutrophil chemotaxis (upper) and NET formation. **(G, H)** Representative immunofluorescent image showing NET formation in an orthotopic HCC mouse model using Hepa1-6 cells with **(H)** or without **(G)** Spp1 overexpression. Scale bar: 50 µm. ns, P > 0.05, *P < 0.05, **P < 0.01, ***P < 0.001, ****P < 0.0001.

Based on data mining of a closely related pro-tumor network between NET score and SPP1 expression level, we further investigated their potential to exert a cooperative effect on cancer behavior. We applied an *in-silico* EMT assay by subtracting the summary of a Z-score-normalized epithelial gene set and a mesenchymal gene set. The EMT scores for the NETs/SPP1-high and -low groups were compared in each cancer type. As expected, almost all cancer types showed significantly higher EMT scores in the NETs/SPP1-high group (**Figure 7A**). For further experimental validation, we knocked down SPP1 expression *via* siRNA in MHCC97H liver cancer cells (**Figures 7B, C**) and then applied NET stimulation. In liver cancer, as reported in our previous work and confirmed by others (8, 30), NETs promote MHCC97H cell invasion in a Transwell assay; however, SPP1 knockdown abrogated the invasion-promoting effect of NETs (**Figures 7D, E**). In an *in vitro* system, we explored how OPN, the SPP1-coding protein, affects neutrophils and found that SPP1 (OPN) promotes neutrophil chemotaxis and OPN directly stimulated NET formation (**Figure 7F**). SPP1 encodes the secretory factor osteopontin (OPN), which has broad immune-associated regulatory functions, especially myeloid cells. As our data mining revealed a tight connection between NET score and SPP1 expression, we hypothesized that SPP1 might also have a regulatory effect on NETs. We used an orthotopic model in immune-competent mice with Hepa1–6 liver cancer cells and Hepa1–6 liver cancer cells overexpressing SPP1 (Hepa1–6^spp1^). We found that Hepa1–6^spp1^-bearing mice had increased NET formation in liver tumors, as revealed by two NET markers, MPO and H3cit expression (**Figures 7G, H**). These results indicated a possible cooperation between SPP1 (OPN) and NETs in promoting cancer cell metastasis.

## Discussion

The presence of neutrophils has been widely recognized in almost all types of solid cancers, and neutrophils have been shown to have both pro- and antitumor effects in different contexts (31). The web-like structures known as neutrophil extracellular traps (NETs) are receiving increasing interest. Most studies have focused on their pro-metastatic effects, especially their roles in regulating tumor proliferation and tumor-associated thrombosis (13, 32, 33). A potential antitumor role of NETs *via* direct cytotoxicity has also been proposed; however, less is known about how NETs may regulate cancer overall.

In this study, we used a widely accepted gene set enrichment analysis (GSEA)-based approach (34, 35) to evaluate features of NETs in 8,059 samples from 22 cancer types. Using a range of methods, we showed that NET score is correlated with key cancer hallmarks, in particular inflammatory responses and EMT (36–38). These findings are consistent with the known function of neutrophils as an innate immune modulator, and many studies have identified metastasis-promoting effects of NETs. However, few studies have explored the effects of NETs on angiogenesis, antiapoptotic pathways, metabolic alterations, and hypoxia in cancer. Although our previous study preliminarily established NETs as an angiogenic regulator in liver cancer (8), the conclusions of this study were far from concrete and complete, and we highlighted the necessity of further experimental validation.

As few studies have examined NETs in the context of the tumor microenvironment, we used MCPcounter, which allowed robust quantification of the absolute abundance of eight immune and two stromal cell populations in heterogeneous tissues from transcriptomic data. The end result of this analysis was the quantification of infiltrating immune cells that might cooperate with NETs in cancer. Neutrophils, of course, were most relevant to NETs, proving the validity of the NET score. Moreover, infiltration of myeloid-derived cells, e.g., macrophages and monocytic cells, was also highly correlated with NET score. By contrast, two types of tumor-killing cells, T cells and NK cells, were less correlated (**Figure S2**), suggesting that NETs might also have an immune suppressive effect in cancer by attracting myeloid cells but not lymphatic cells. Consistent with our analysis, we confirmed that the NET score is positively correlated with M1 macrophage polarization in lung tissue during acute respiratory distress syndrome (39). NETs can prime macrophages for cytokine release (40), while on the other hand, NETs can inhibit T cell- and NK cell-dependent killing by physically coating tumor cells (17). Based on these results, we will focus more attention on the interaction between NETs and immune cells in our further research.

In this study, we also explored the relationship between NET score and the response to anti-PD1 therapy in skin cancer and bladder cancer patients. We found a slight but significant decrease in NET score in patients that responded to PD1 blockade (**Figure 2C**), indicating that NETs negatively regulate T cell-induced cytotoxicity. These observations are consistent with the recent finding that IL17-induced NET formation excludes cytotoxic CD8+ T cells from tumors (18). Together, these findings suggest that NET inhibition increases sensitivity to PD1 therapy.

The prognostic impact of the NET score varied in different contexts, with a tendency toward a poor prognostic value (41–43). Previous studies have revealed a connection between NETs and poor prognosis in glioma (29), large B-cell lymphoma (44), esophageal cancer (45), and gastric adenocarcinoma (42) and a connection to favorable prognosis in high-grade serious ovarian cancer (19). In our study, NET-related effects on survival varied as the NET score predicted a better prognosis in 10 cancer types but a poorer prognosis in 9 cancer type. Two factors might contribute to the prognostic diversity of NETs in various cancer types. The first possibility is that NETs were quantified with different methods in different cancer types. The majority of studies on individual cancer types used circulating markers, such as the MPO–DNA complex, as a NET marker, while in this study we used a pan-cancer tumor-infiltrated NET score derived from a broad data set. These differences might have led to methodology-dependent variation across different cancer types. The second possibility is the number of markers analyzed. Due to experimental constraints, most studies only analyze two or three NET-associated proteins as NET markers, e.g., MPO or DNA levels. Perhaps this narrow view is insufficient to provide a more global perspective. In this study, we estimated NET enrichment based on 23 major NET-associated genes, which likely led to more accurate conclusions. Moreover, potential minor but important responses of various malignancies to NETs are also worth considering. We suggest placing more emphasis on the prognostic power of tumor-infiltrated NETs in further studies.

The allocation of cancer types into groups with poor or favorable NET-related survival enabled the exploration of shared and exclusive NET-related biological modification patterns across various cancer types. We applied a novel strategy that allowed us to identify NET-related genes that contribute to cancer progression. We found genes related to extracellular matrix organization, such as SPP1, MT1L, and COL3A1 (among others), which functioned as NET-associated regulatory genes exclusively in cancer types with poor NET-related survival. These genes are well known to promote cancer metastasis, cancer growth, and cancer aggressiveness (10, 46–48). Until now, there have been no reports on how NETs modulate the extracellular matrix in cancer, and further research on this topic is urgently required.

SPP1, whose protein product is known as OPN, is closely associated with tumorigenesis and metastasis in several cancer types. SPP1 also modulates the TME by functioning as a chemotaxis factor for various immune cells, including neutrophils, macrophages, and dendritic cells; however, to the best of our knowledge a role for SPP1 as a NET stimulator in cancer has not yet been identified. Interestingly, Kasetty et al. reported an inhibitory effect of SPP1 on NET formation after lung injury (49). Our study reveals a strong positive correlation between SPP1 and NETs in multiple cancer types, and co-upregulation of SPP1 expression and NET score were related to higher EMT scores in all examined cancer types. In addition, SPP1 knockdown attenuated NET-induced migration in cancer cell lines. These results indicated that at least to some degree, NET-induced cancer metastasis is SPP1 dependent.

In summary, *via* an integrated analysis, we now provide a new and comprehensive perspective on the dysregulatory role of NETs in cancer. We also raise doubt regarding the pro- or anti-tumorigenic roles of NETs. We believe that in addition to the well-illustrated role of NETs in promoting malignancy there is also a NET-associated antitumor response. The identification of this anti-tumorigenic response might support the development of new therapeutic strategies that harness the anticancer effects of NETs. A potential strategy would be to leverage the effect of NETs as an inflammatory regulator to ignite the inflammatory response in “cold” tumor zones, thereby sensitizing the cancer cells to immune checkpoint therapy. Moreover, we have also provided new insight into how NETs are linked to other cancer hallmarks, cancer prognosis, and several gene sets. Among these new connections, the NET-induced inflammatory response as well as the effects of NETs on immune cell chemotaxis and chemotaxis factor regulation are particularly worthy of further investigation.

## Data Availability Statement

The original contributions presented in the study are included in the article/**Supplementary Material**. Further inquiries can be directed to the corresponding authors.

## Ethics Statement

The animal study was reviewed and approved by the Department of Laboratory of Animal Science Fudan University.

## Author Contributions

All authors contributed to the work presented in this paper. Conceptualization: L-XQ, L-YY. GEO and TCGA resources, data analysis, visualization, and validation: X-TS and S-ZX. Writing—original draft preparation: X-TS and S-ZX. Writing—editing: JX and X-TS. Statistical analysis: X-TS and S-ZX. Supervision: L-XQ and L-YY. All authors contributed to the article and approved the submitted version.

## Funding

This work was jointly supported by the National Natural Science Foundation of China (No. 81672820 and No. 81930074 and No. 91959203 to L-XQ, 82002532 to L-YY), the Sailing Program of the Shanghai Science and Technology Committee (19YF1405000), and the “Fuqing Scholar” Student Scientific Research Program of Shanghai Medical College (FQXZ202115B).

## Conflict of Interest

The authors declare that the work was conducted in the absence of any commercial or financial relationships that could be construed as a potential conflict of interest.

## Publisher’s Note

All claims expressed in this article are solely those of the authors and do not necessarily represent those of their affiliated organizations, or those of the publisher, the editors and the reviewers. Any product that may be evaluated in this article, or claim that may be made by its manufacturer, is not guaranteed or endorsed by the publisher.

## Supplementary Material

Supplementary Table 1 Cancer samples included in this study.

Supplementary Figure 1 Pearson correlations between protein and transcription levels of NET-related genes in BRCA.

Supplementary Figure 2 Pearson correlation between ssGSEA enrichment scores of a hallmark gene set and NETs score in cancers.

Supplementary Figure 3 Pearson correlation between MCPcounter enrichment score of 10 immune cells **(A-C)** or immune checkpoint **(D)** and NETs score in cancers.

Supplementary Figure 4 Volcano plot showing the upregulation of chemotaxis genes in the high-NETs group.

Supplementary Figure 5 Kaplan-Meier curve showing the prognosis difference between the NETs-high and NET-low groups in cancers.

Supplementary Figure 6
**(A, B)** GSEA analysis of 2 NETs-associated regulatory gene sets between cancers with favorable or risk NETs-related survival. **(C)** The HR distribution of NETs-associated regulatory genes in cancers. Red indicates risk genes, while blue indicates favorable genes.

## Abbreviations

NETs, neutrophil extracellular traps; ECM, extracellular matrix; ICGs, immune checkpoint genes; EMT, epithelial mesenchymal transition

## References

[1] Kolaczkowska E Kubes P. Neutrophil Recruitment and Function in Health and Inflammation. Nat Rev Immunol (2013) 13:159–75. doi: 10.1038/nri3399 23435331

[2] Erpenbeck L Schon MP. Neutrophil Extracellular Traps: Protagonists of Cancer Progression? Oncogene (2017) 36:2483–90. doi: 10.1038/onc.2016.406 27941879

[3] Gardinassi LG Desouza-Vieira TS Da Silva NO Garcia GR Borges VM Campos RNS. Molecular Signatures of Neutrophil Extracellular Traps in Human Visceral Leishmaniasis. Parasit Vectors (2017) 10:285. doi: 10.1186/s13071-017-2222-5 28583201 PMC5460406

[4] Wither JE Prokopec SD Noamani B Chang NH Bonilla D Touma Z. Identification of a Neutrophil-Related Gene Expression Signature That Is Enriched in Adult Systemic Lupus Erythematosus Patients With Active Nephritis: Clinical/pathologic Associations and Etiologic Mechanisms. PloS One (2018) 13:e0196117. doi: 10.1371/journal.pone.0196117 29742110 PMC5942792

[5] Laridan E Martinod K De Meyer SF. Neutrophil Extracellular Traps in Arterial and Venous Thrombosis. Semin Thromb Hemost (2019) 45:86–93. doi: 10.1055/s-0038-1677040 30634198

[6] Jorch SK Kubes P. An Emerging Role for Neutrophil Extracellular Traps in Noninfectious Disease. Nat Med (2017) 23:279–87. doi: 10.1038/nm.4294 28267716

[7] Liew PX Kubes P. The Neutrophil’s Role During Health and Disease. Physiol Rev (2019) 99:1223–48. doi: 10.1152/physrev.00012.2018 30758246

[8] Yang LY Luo Q Lu L Zhu WW Sun HT Wei R. Increased Neutrophil Extracellular Traps Promote Metastasis Potential of Hepatocellular Carcinoma *via* Provoking Tumorous Inflammatory Response. J Hematol Oncol (2020) 13:3. doi: 10.1186/s13045-019-0836-0 31907001 PMC6945602

[9] Hong IS. Stimulatory Versus Suppressive Effects of GM-CSF on Tumor Progression in Multiple Cancer Types. Exp Mol Med (2016) 48:e242. doi: 10.1038/emm.2016.64 27364892 PMC4973317

[10] Zhu Y Yang J Xu D Gao XM Zhang Z Hsu JL. Disruption of Tumour-Associated Macrophage Trafficking by the Osteopontin-Induced Colony-Stimulating Factor-1 Signalling Sensitises Hepatocellular Carcinoma to Anti-PD-L1 Blockade. Gut (2019) 68:1653–66. doi: 10.1136/gutjnl-2019-318419 30902885

[11] Demers M Krause DS Schatzberg D Martinod K Voorhees JR Fuchs TA. Cancers Predispose Neutrophils to Release Extracellular DNA Traps That Contribute to Cancer-Associated Thrombosis. Proc Natl Acad Sci USA (2012) 109:13076–81. doi: 10.1073/pnas.1200419109 PMC3420209 22826226

[12] Snoderly HT Boone BA Bennewitz MF. Neutrophil Extracellular Traps in Breast Cancer and Beyond: Current Perspectives on NET Stimuli, Thrombosis and Metastasis, and Clinical Utility for Diagnosis and Treatment. Breast Cancer Res (2019) 21:145. doi: 10.1186/s13058-019-1237-6 31852512 PMC6921561

[13] Yang L Liu Q Zhang X Liu X Zhou B Chen J. DNA of Neutrophil Extracellular Traps Promotes Cancer Metastasis *via* CCDC25. Nature (2020) 583:133–8. doi: 10.1038/s41586-020-2394-6 32528174

[14] Xiao Y Cong M Li J He D Wu Q Tian P. Cathepsin C Promotes Breast Cancer Lung Metastasis by Modulating Neutrophil Infiltration and Neutrophil Extracellular Trap Formation. Cancer Cell (2021) 39:423–437 e427. doi: 10.1016/j.ccell.2020.12.012 33450198

[15] Albrengues J Shields MA Ng D Park CG Ambrico A Poindexter ME. Neutrophil Extracellular Traps Produced During Inflammation Awaken Dormant Cancer Cells in Mice. Science (2018) 361:eaao4227. doi: 10.1126/science.aao4227 30262472 PMC6777850

[16] Yazdani HO Roy E Comerci AJ van der Windt DJ Zhang H Huang H. Neutrophil Extracellular Traps Drive Mitochondrial Homeostasis in Tumors to Augment Growth. Cancer Res (2019) 79:5626–39. doi: 10.1158/0008-5472.CAN-19-0800 PMC6825588 31519688

[17] Teijeira A Garasa S Gato M Alfaro C Migueliz I Cirella A. CXCR1 and CXCR2 Chemokine Receptor Agonists Produced by Tumors Induce Neutrophil Extracellular Traps That Interfere With Immune Cytotoxicity. Immunity (2020) 52:856–871 e858. doi: 10.1016/j.immuni.2020.03.001 32289253

[18] Zhang Y Chandra V Riquelme Sanchez E Dutta P Quesada PR Rakoski A. Interleukin-17-Induced Neutrophil Extracellular Traps Mediate Resistance to Checkpoint Blockade in Pancreatic Cancer. J Exp Med (2020) 217:e20190354. doi: 10.1084/jem.20190354 32860704 PMC7953739

[19] Muqaku B Pils D Mader JC Aust S Mangold A Muqaku L. Neutrophil Extracellular Trap Formation Correlates With Favorable Overall Survival in High Grade Ovarian Cancer. Cancers (Basel) (2020) 12:505. doi: 10.3390/cancers12020505 PMC7072166 32098278

[20] Schedel F Mayer-Hain S Pappelbaum KI Metze D Stock M Goerge T. Evidence and Impact of Neutrophil Extracellular Traps in Malignant Melanoma. Pigment Cell Melanoma Res (2020) 33:63–73. doi: 10.1111/pcmr.12818 31402559

[21] Liu K Sun E Lei M Li L Gao J Nian X. BCG-Induced Formation of Neutrophil Extracellular Traps Play an Important Role in Bladder Cancer Treatment. Clin Immunol (2019) 201:4–14. doi: 10.1016/j.clim.2019.02.005 30771501

[22] O’donoghue AJ Jin Y Knudsen GM Perera NC Jenne DE Murphy JE. Global Substrate Profiling of Proteases in Human Neutrophil Extracellular Traps Reveals Consensus Motif Predominantly Contributed by Elastase. PloS One (2013) 8:e75141. doi: 10.1371/journal.pone.0075141 24073241 PMC3779220

[23] Hanzelmann S Castelo R Guinney J. GSVA: Gene Set Variation Analysis for Microarray and RNA-Seq Data. BMC Bioinf (2013) 14:7. doi: 10.1186/1471-2105-14-7 PMC3618321 23323831

[24] Becht E Giraldo NA Lacroix L Buttard B Elarouci N Petitprez F. Estimating the Population Abundance of Tissue-Infiltrating Immune and Stromal Cell Populations Using Gene Expression. Genome Biol (2016) 17:218. doi: 10.1186/s13059-016-1070-5 27765066 PMC5073889

[25] Hugo W Zaretsky JM Sun L Song C Moreno BH Hu-Lieskovan S. Genomic and Transcriptomic Features of Response to Anti-PD-1 Therapy in Metastatic Melanoma. Cell (2016) 165:35–44. doi: 10.1016/j.cell.2016.02.065 26997480 PMC4808437

[26] Riaz N Havel JJ Makarov V Desrichard A Urba WJ Sims JS. Tumor and Microenvironment Evolution During Immunotherapy With Nivolumab. Cell (2017) 171:934–949 e916. doi: 10.1016/j.cell.2017.09.028 29033130 PMC5685550

[27] Snyder A Nathanson T Funt SA Ahuja A Buros Novik J Hellmann MD. Contribution of Systemic and Somatic Factors to Clinical Response and Resistance to PD-L1 Blockade in Urothelial Cancer: An Exploratory Multi-Omic Analysis. PloS Med (2017) 14:e1002309. doi: 10.1371/journal.pmed.1002309 28552987 PMC5446110

[28] Zhou SL Dai Z Zhou ZJ Wang XY Yang GH Wang Z. Overexpression of CXCL5 Mediates Neutrophil Infiltration and Indicates Poor Prognosis for Hepatocellular Carcinoma. Hepatology (2012) 56:2242–54. doi: 10.1002/hep.25907 22711685

[29] Zha C Meng X Li L Mi S Qian D Li Z. Neutrophil Extracellular Traps Mediate the Crosstalk Between Glioma Progression and the Tumor Microenvironment *via* the HMGB1/RAGE/IL-8 Axis. Cancer Biol Med (2020) 17:154–68. doi: 10.20892/j.issn.2095-3941.2019.0353 PMC7142852 32296583

[30] Yang L Liu L Zhang R Hong J Wang Y Wang J. IL-8 Mediates a Positive Loop Connecting Increased Neutrophil Extracellular Traps (NETs) and Colorectal Cancer Liver Metastasis. J Cancer (2020) 11:4384–96. doi: 10.7150/jca.44215 PMC7255375 32489457

[31] Giese MA Hind LE Huttenlocher A. Neutrophil Plasticity in the Tumor Microenvironment. Blood (2019) 133:2159–67. doi: 10.1182/blood-2018-11-844548 PMC6524564 30898857

[32] Huang H Zhang H Onuma AE Tsung A. Neutrophil Elastase and Neutrophil Extracellular Traps in the Tumor Microenvironment. Adv Exp Med Biol (2020) 1263:13–23. doi: 10.1007/978-3-030-44518-8_2 32588320 PMC11770835

[33] Masucci MT Minopoli M Del Vecchio S Carriero MV. The Emerging Role of Neutrophil Extracellular Traps (NETs) in Tumor Progression and Metastasis. Front Immunol (2020) 11:1749. doi: 10.3389/fimmu.2020.01749 33042107 PMC7524869

[34] Chakravarthy A Khan L Bensler NP Bose P De Carvalho DD. TGF-Beta-Associated Extracellular Matrix Genes Link Cancer-Associated Fibroblasts to Immune Evasion and Immunotherapy Failure. Nat Commun (2018) 9:4692. doi: 10.1038/s41467-018-06654-8 30410077 PMC6224529

[35] Shen X Hu B Xu J Qin W Fu Y Wang S. The M6a Methylation Landscape Stratifies Hepatocellular Carcinoma Into 3 Subtypes With Distinct Metabolic Characteristics. Cancer Biol Med (2020) 17:937–52. doi: 10.20892/j.issn.2095-3941.2020.0402 PMC7721089 33299645

[36] Aldabbous L Abdul-Salam V Mckinnon T Duluc L Pepke-Zaba J Southwood M. Neutrophil Extracellular Traps Promote Angiogenesis: Evidence From Vascular Pathology in Pulmonary Hypertension. Arterioscler Thromb Vasc Biol (2016) 36:2078–87. doi: 10.1161/ATVBAHA.116.307634 27470511

[37] Martins-Cardoso K Almeida VH Bagri KM Rossi MID Mermelstein CS Konig S. Neutrophil Extracellular Traps (NETs) Promote Pro-Metastatic Phenotype in Human Breast Cancer Cells Through Epithelial-Mesenchymal Transition. Cancers (Basel) (2020) 12:1542. doi: 10.3390/cancers12061542 PMC7352979 32545405

[38] Zhu T Zou X Yang C Li L Wang B Li R. Neutrophil Extracellular Traps Promote Gastric Cancer Metastasis by Inducing Epithelialmesenchymal Transition. Int J Mol Med (2021) 48:127. doi: 10.3892/ijmm.2021.4960 34013374 PMC8128417

[39] Song C Li H Li Y Dai M Zhang L Liu S. NETs Promote ALI/ARDS Inflammation by Regulating Alveolar Macrophage Polarization. Exp Cell Res (2019) 382:111486. doi: 10.1016/j.yexcr.2019.06.031 31255598

[40] Warnatsch A Ioannou M Wang Q Papayannopoulos V. Inflammation. Neutrophil Extracellular Traps License Macrophages for Cytokine Production in Atherosclerosis. Science (2015) 349:316–20. doi: 10.1126/science.aaa8064 PMC4854322 26185250

[41] Grilz E Mauracher LM Posch F Konigsbrugge O Zochbauer-Muller S Marosi C. Citrullinated Histone H3, a Biomarker for Neutrophil Extracellular Trap Formation, Predicts the Risk of Mortality in Patients With Cancer. Br J Haematol (2019) 186:311–20. doi: 10.1111/bjh.15906 PMC6618331 30968400

[42] Zhang Y Hu Y Ma C Sun H Wei X Li M. Diagnostic, Therapeutic Predictive, and Prognostic Value of Neutrophil Extracellular Traps in Patients With Gastric Adenocarcinoma. Front Oncol (2020) 10:1036. doi: 10.3389/fonc.2020.01036 32714865 PMC7344202

[43] Rosell A Aguilera K Hisada Y Schmedes C Mackman N Wallen H. Prognostic Value of Circulating Markers of Neutrophil Activation, Neutrophil Extracellular Traps, Coagulation and Fibrinolysis in Patients With Terminal Cancer. Sci Rep (2021) 11:5074. doi: 10.1038/s41598-021-84476-3 33658563 PMC7930088

[44] Nie M Yang L Bi X Wang Y Sun P Yang H. Neutrophil Extracellular Traps Induced by IL8 Promote Diffuse Large B-Cell Lymphoma Progression *via* the TLR9 Signaling. Clin Cancer Res (2019) 25:1867–79. doi: 10.1158/1078-0432.CCR-18-1226 30446590

[45] Zhang H Lv H Weng M Wang H Cata JP Chen W. Preoperative Leukocytosis Is Associated With Increased Tumor-Infiltrating Neutrophil Extracellular Traps and Worse Outcomes in Esophageal Cancer. Ann Transl Med (2020) 8:441. doi: 10.21037/atm.2020.03.190 32395485 PMC7210211

[46] Zhu Y Gao XM Yang J Xu D Zhang Y Lu M. C-C Chemokine Receptor Type 1 Mediates Osteopontin-Promoted Metastasis in Hepatocellular Carcinoma. Cancer Sci (2018) 109:710–23. doi: 10.1111/cas.13487 PMC5834777 29285854

[47] Xu S Xu H Wang W Li S Li H Li T. The Role of Collagen in Cancer: From Bench to Bedside. J Transl Med (2019) 17:309. doi: 10.1186/s12967-019-2058-1 31521169 PMC6744664

[48] Zunder SM Gelderblom H Tollenaar RA Mesker WE. The Significance of Stromal Collagen Organization in Cancer Tissue: An in-Depth Discussion of Literature. Crit Rev Oncol Hematol (2020) 151:102907. doi: 10.1016/j.critrevonc.2020.102907 32408009

[49] Kasetty G Papareddy P Bhongir RKV Ali MN Mori M Wygrecka M. Osteopontin Protects Against Lung Injury Caused by Extracellular Histones. Mucosal Immunol (2019) 12:39–50. doi: 10.1038/s41385-018-0079-3 30115999

