# Supplementary material for: Pan-Cancer Analysis Reveals a Distinct Neutrophil Extracellular Trap-Associated Regulatory Pattern
Source: Front Immunol. 2022 Mar 31;13:798022. doi: 10.3389/fimmu.2022.798022 (PMC9009150; doi:10.3389/fimmu.2022.798022)
Supplement: Supplementary Table 1 — Cancer samples included in this study. [file DataSheet_1.zip › FigS4.pdf]

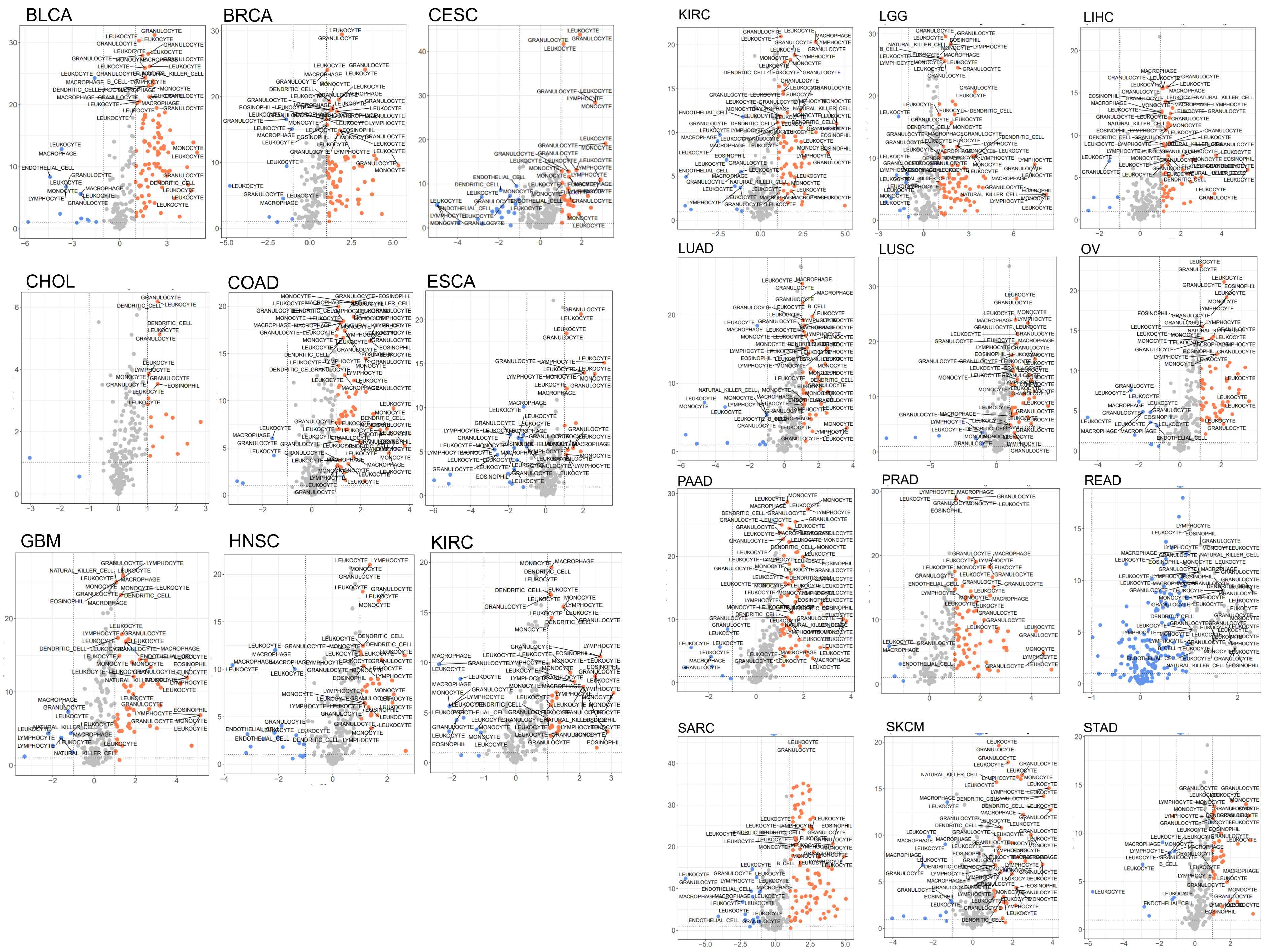
BLCA
BRCA
CESC
CHOL
COAD
ESCA
GBM
HNSC
KIRC
KIRC
LGG
LIHC
LUAD
LUSC
OV
PAAD
PRAD
READ
SARC
SKCM
STAD
