# Supplementary figures and images for: Pan-Cancer Analysis Reveals a Distinct Neutrophil Extracellular Trap-Associated Regulatory Pattern
Source: Front Immunol. 2022 Mar 31;13:798022. doi: 10.3389/fimmu.2022.798022 (PMC9009150; doi:10.3389/fimmu.2022.798022)

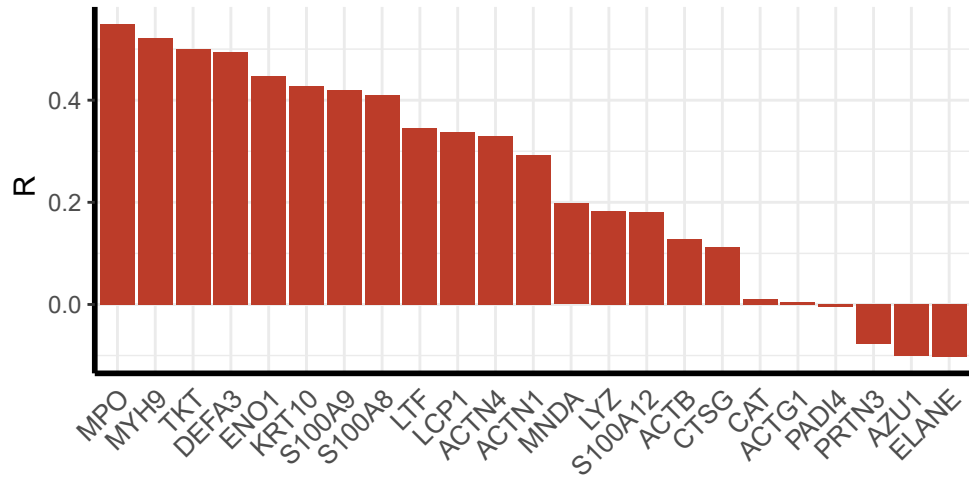

Supplement: Supplementary Table 1 — Cancer samples included in this study. [file DataSheet_1.zip › FigS1.pdf]

Pearson R

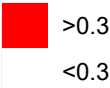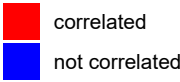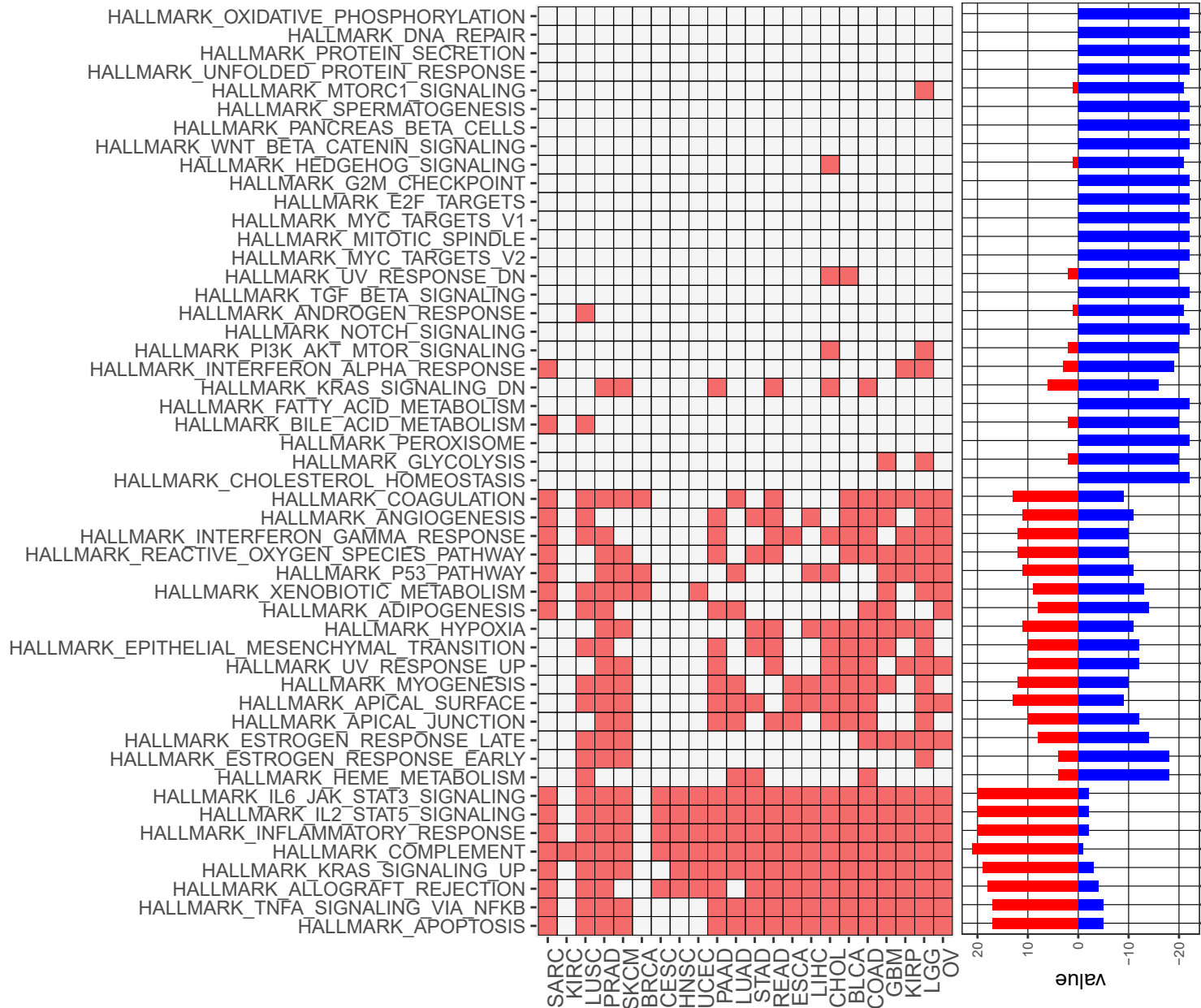

.....

Supplement: Supplementary Table 1 — Cancer samples included in this study. [file DataSheet_1.zip › FigS2.pdf]

A

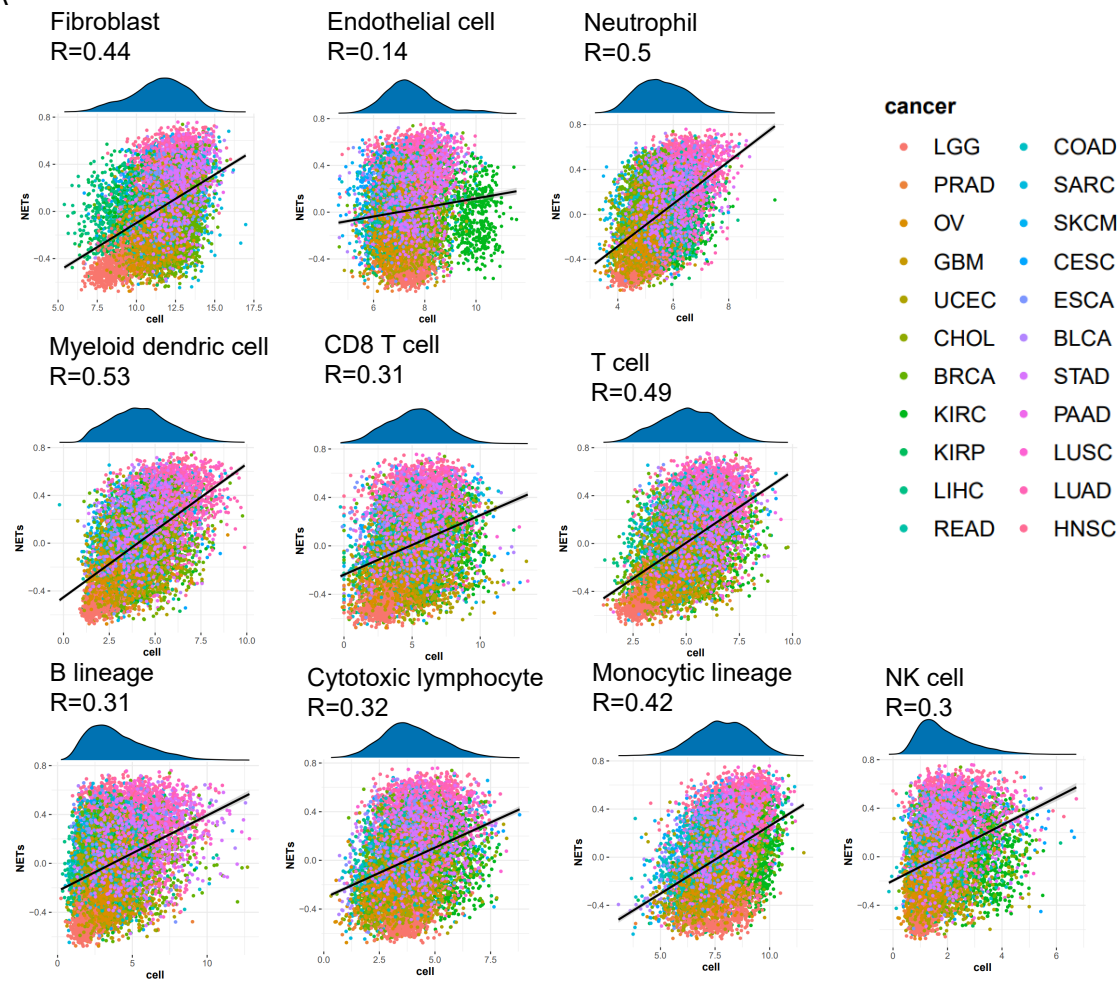

B

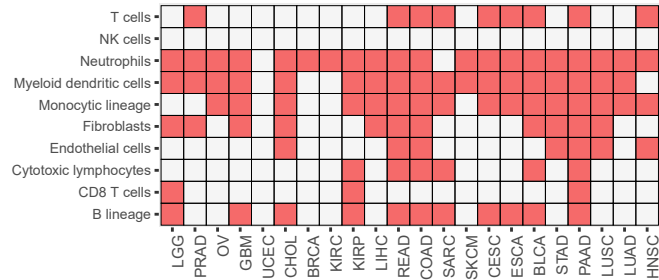

C

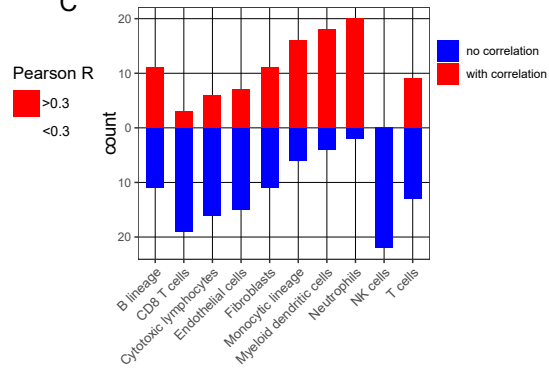

D

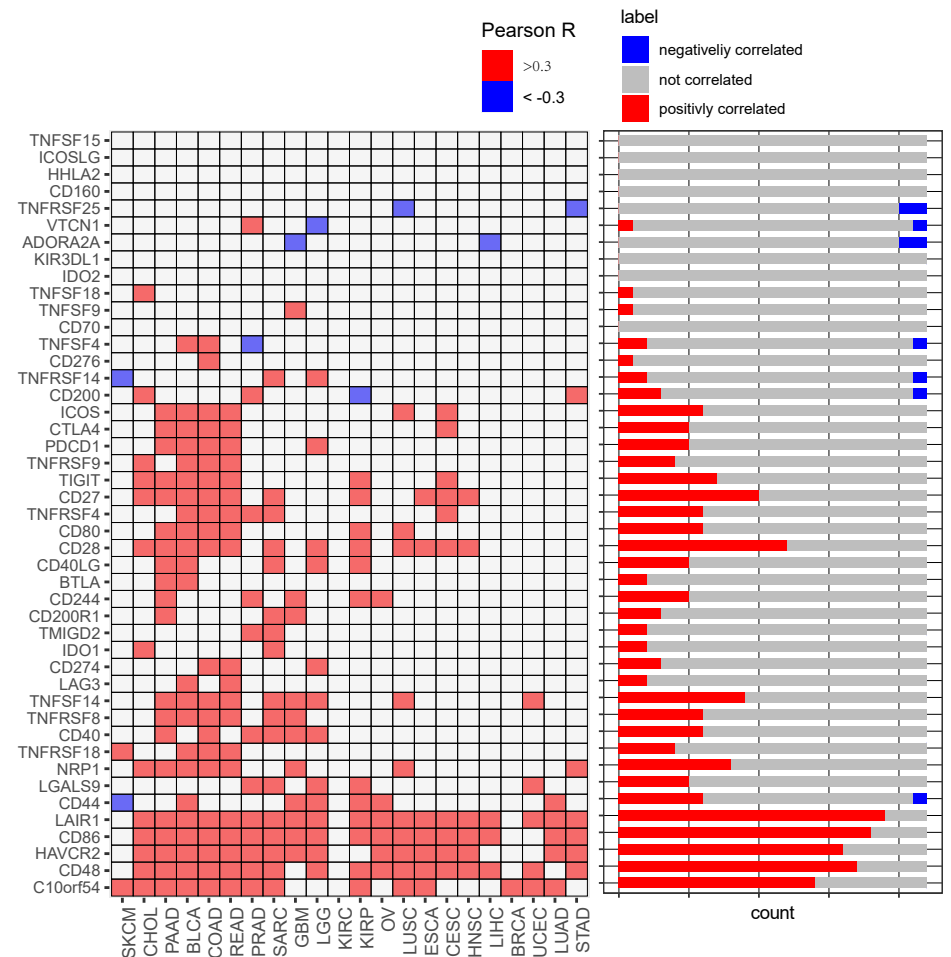

Supplement: Supplementary Table 1 — Cancer samples included in this study. [file DataSheet_1.zip › FigS3.pdf]

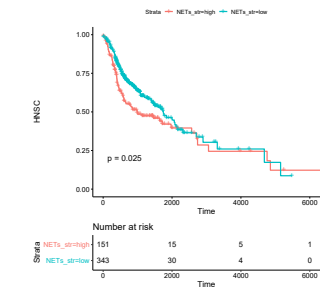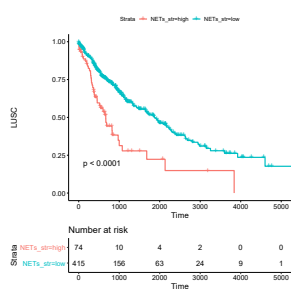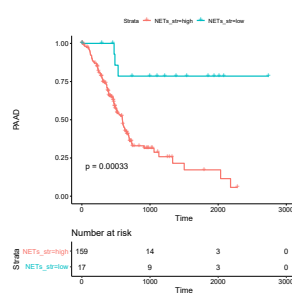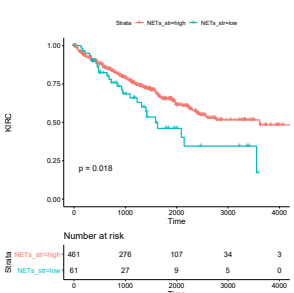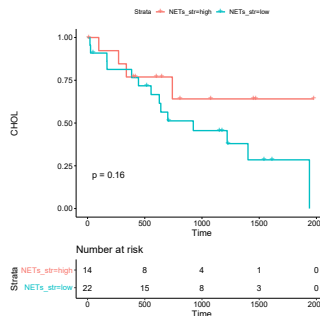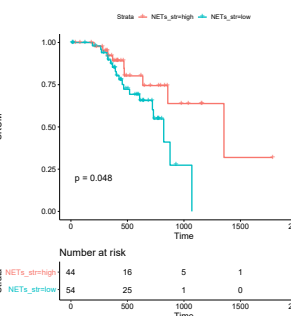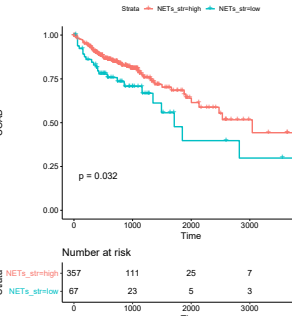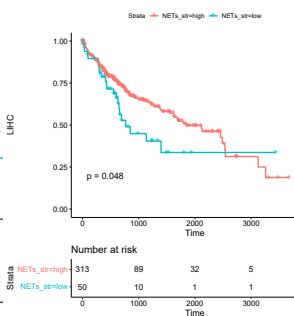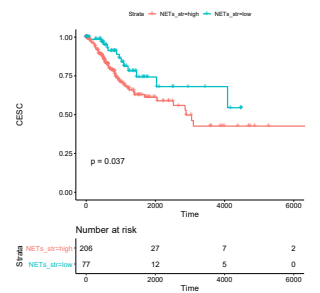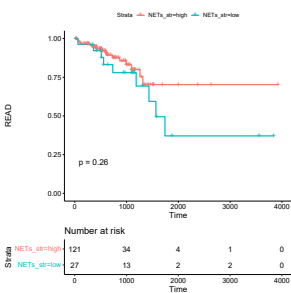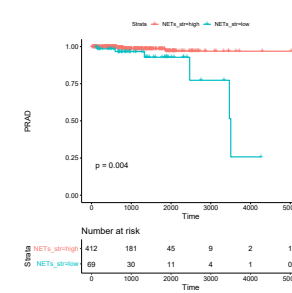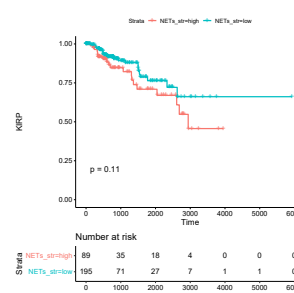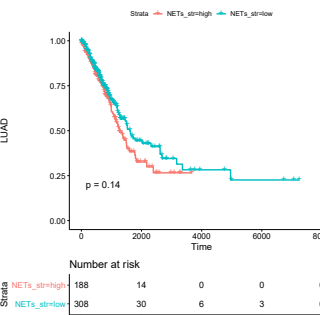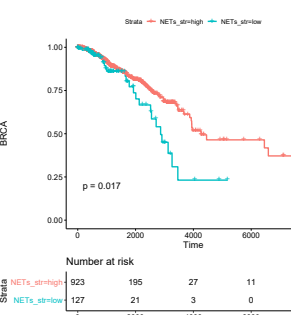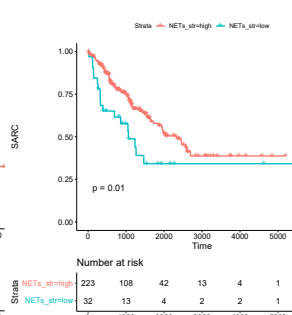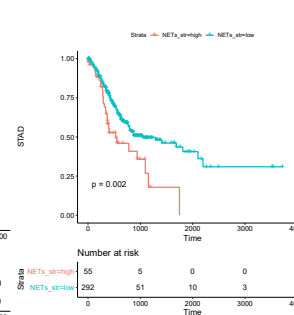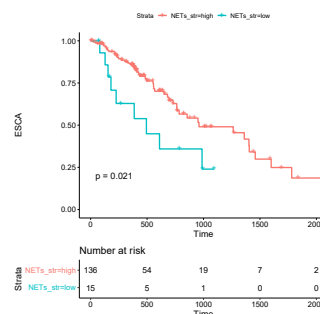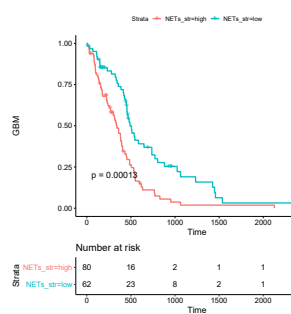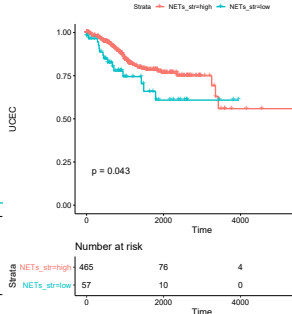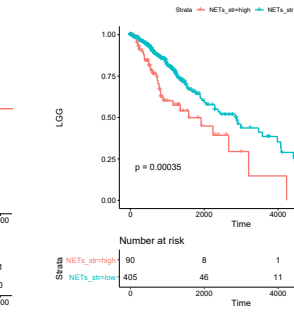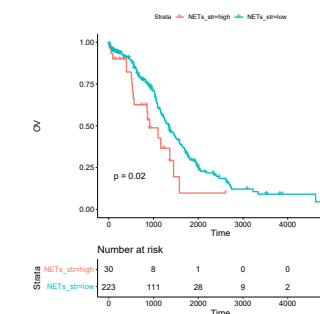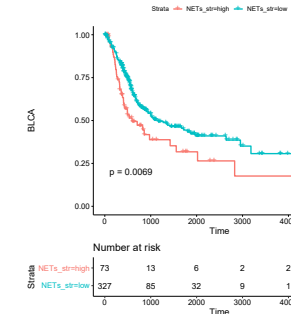

Supplement: Supplementary Table 1 — Cancer samples included in this study. [file DataSheet_1.zip › FigS5.pdf]

A

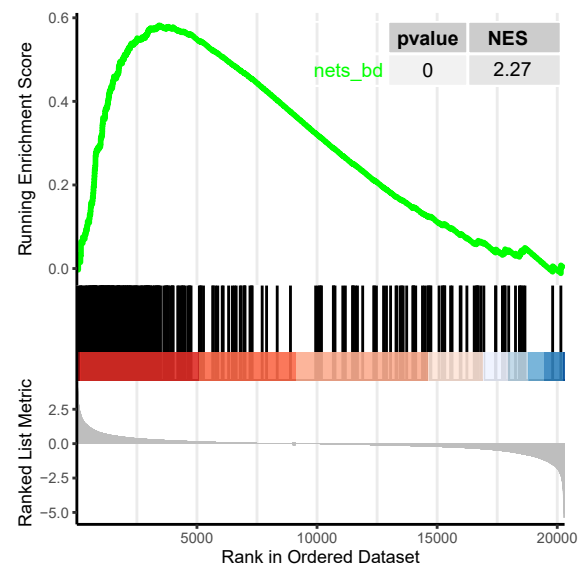

B

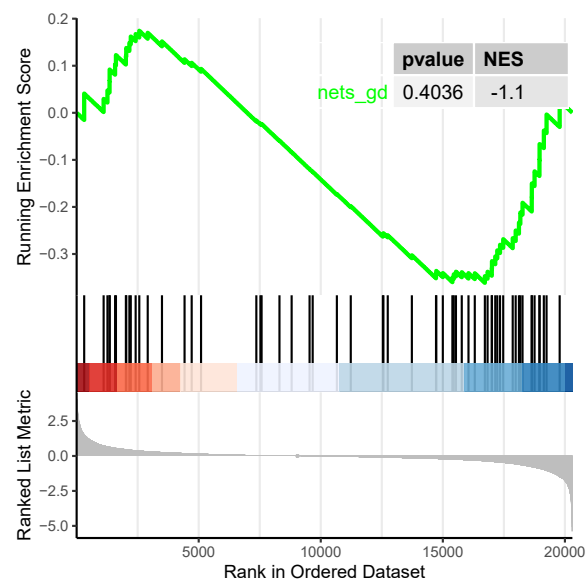

C

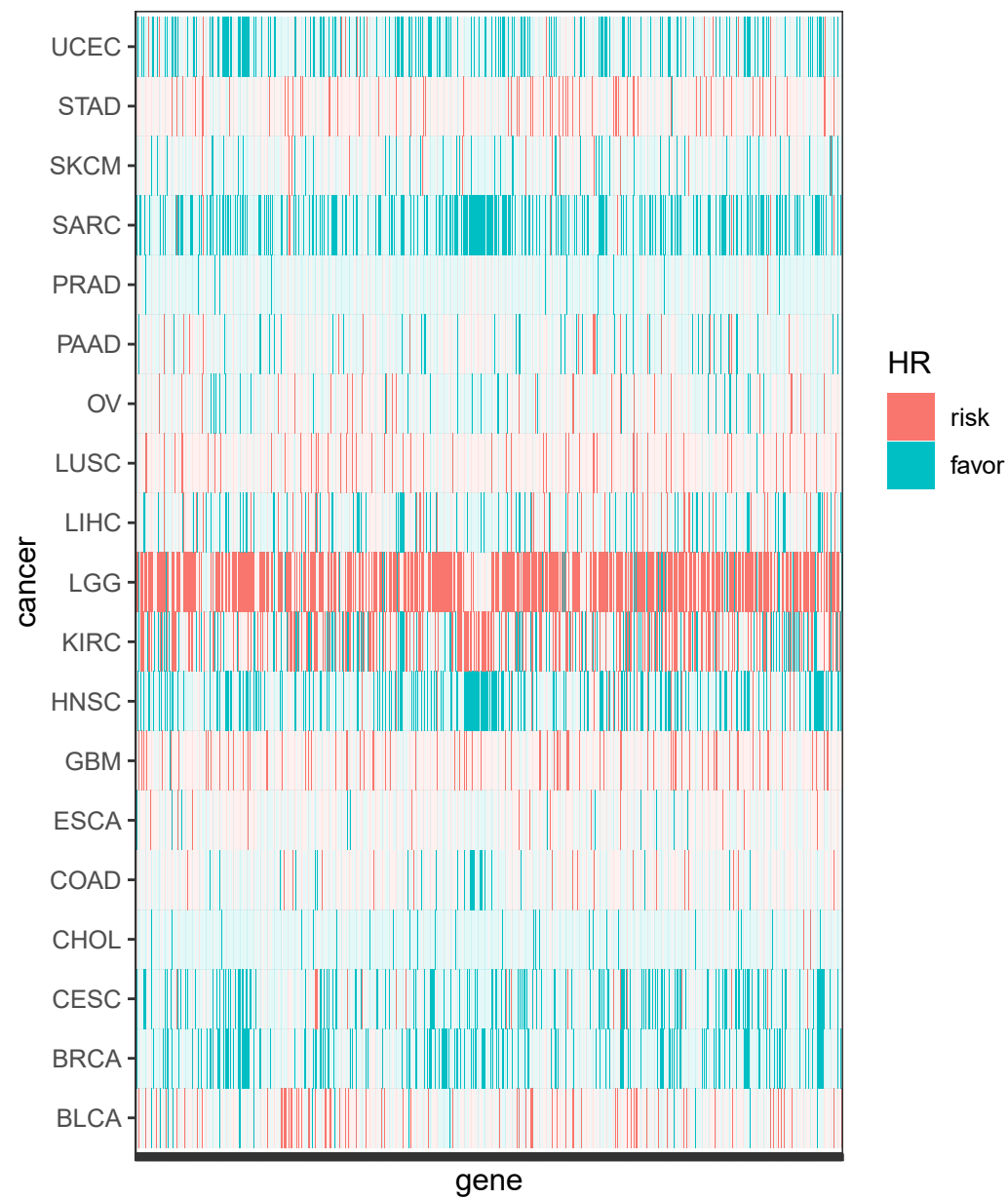

Supplement: Supplementary Table 1 — Cancer samples included in this study. [file DataSheet_1.zip › FigS6.pdf]
